# Sexual and Reproductive Health Education for Youth with Intellectual Disabilities: a Mixed Methods Study of Professionals’ Practices and Needs

**DOI:** 10.1007/s11121-023-01522-4

**Published:** 2023-06-08

**Authors:** Lisa Colarossi, Kate L. Collier, Randa Dean, Siana Pérez, Marlene O. Riquelme

**Affiliations:** Planned Parenthood of Greater New York, New York, NY, USA

**Keywords:** Sexual health, Intellectual disabilities, Formative research, Educational materials

## Abstract

We conducted formative research to inform the creation of innovative new tools and strategies to engage professionals in communicating with youth with intellectual disabilities about sexual health. The research was guided by a multidisciplinary network of experts and an advisory board of self-advocates with intellectual disabilities and caregivers that make up Project SHINE: the Sexual Health Innovation Network for Equitable Education. A cross-sectional mixed-methods study utilized survey data from 632 disability support professionals who provide services to youth ages 16–24 with intellectual disabilities (ID). We then conducted focus groups with 36 professionals to obtain more in-depth information related to organizational support needs and suitable contexts, methods, and tools for sexuality education. Participants included licensed/credentialed direct service professionals (social workers, nurses, teachers), non-licensed direct service providers (case managers, supportive care specialists, residential care line staff), and program administrators. Quantitative and qualitative data analyses triangulated the findings across four content areas: attitudes about providing sexual health information to youth with ID, preparedness to communicate about sexuality, current communication practices, and professional needs in the field for new teaching tools and methods. We discuss how findings can be used to guide the creation and successful implementation of innovative new sexual health learning tools for youth with intellectual disabilities.

## Introduction

Sexuality education resources that address the diverse learning needs of adolescents and young adults with intellectual disabilities (ID) are greatly needed. As a normal part of growth and development, youth and adults with ID experience sexual desires and behaviors as diverse as that of the broader population (Gomez, 2012; Isler et al., 2009). Research demonstrates that youth with ID are less likely than youth without ID to engage in safer sex practices (Cheng & Udry, 2005; Isler et al., 2009) and in turn are at increased risk for sexually transmitted infections (STIs) (Mandell et al., 2008) and pregnancy (Baines et al., 2018). Youth with ID are also disproportionately impacted by sexual abuse (McDaniels & Fleming, 2016), and have more limited access to resources for associated trauma. A meta-analysis by Jones and colleagues (2012) estimated that the risk of sexual violence was nearly five times greater for youth with ID than for those without. Yet, one harmful myth is that people with ID only have abusive sexual relationships as victims or perpetrators of violence (Kayser et al., 2018). Youth with ID can and do have fulfilling romantic and sexual relationships (Retznik et al., 2021).

A key driver for inequities in sexual health outcomes for youth with and without ID is access to suitable sexual health information, education, and care (McDaniels & Fleming, 2016). Developmentally appropriate sex education can reinforce the rights of youth with ID to make informed choices and participate in their communities (Gardiner, 2018), yet people with ID report wanting more sexual health information than they currently have (Addlakha et al., 2017). Advocacy work led by people with disabilities has resulted in more integrated learning environments for people with and without ID, although the specific needs of youth with ID are not always met in these environments (Moring, 2019). There are few sex education materials for youth with ID, and those that exist have not been assessed for efficacy or culturally appropriate and trauma-informed standards (Schaafsma et al., 2015; SHEIDD Project, 2019).

Given the significant interface with social services for many young people with ID, the knowledge, attitudes, and behaviors of social service professionals can impact youths’ receipt of sexual and reproductive health (SRH) information, education, and care (Moring, 2019). Several studies suggest that a majority of disability support professionals do not receive specific training related to sexuality (McConkey & Ryan, 2001; Meaney-Tavares & Gavidia-Payne, 2012). In a survey of 163 professionals, 61% indicated they do not provide sex education (Schaafsma et al., 2014). Professionals who provided sex education rated their environments as being more conducive to sex education, were more satisfied with their own capacity to convey information, had more colleagues who also taught sex education, and were more likely to have educational materials in their work setting. Those who did not teach sex education said that their clients are not sexually active and/or do not want such education, and that they do not have the requisite professional knowledge and skills. Further, across diverse national contexts, professionals’ attitudes toward sexuality among people with ID have been mixed, with some studies finding attitudes that affirm the sexuality of people with ID (Bazzo et al., 2007; Gilmore & Chambers, 2010), while others found restrictive or disapproving attitudes (Tamas et al., 2019; Young et al., 2012).

To better understand professionals’ current communication practices, attitudes, and needs related to sexual and reproductive health (SRH) education for adolescents and young adults with ID, we conducted formative research for a broader initiative, Project SHINE (Sexual Health Innovation Network for Equitable Education – with Youth with Intellectual Disabilities).

### Project SHINE Description and Population of Focus

Project SHINE was created by a network of providers who serve high economic need and racially/ethnically diverse youth with ID and their guardians. It is a 3-year initiative to create and test innovative sex education tools for young adults aged 16–24 years with mild to moderate intellectual disabilities (ID) as defined by the American Psychiatric Association’s (2013). ID is estimated to occur in 2.5% of the population, with the vast majority falling (95%) into the mild to moderate range (National Academies of Sciences, Engineering, and Medicine et al., 2015). People with mild to moderate ID have abilities to communicate and socially function with limited to moderate ongoing support, while people with severe to profound ID have limited to no communication skills and require extensive to pervasive support. We chose to focus on young adults with mild to moderate ID because they are well suited to utilize SRH information and services, and benefit from health interventions that require some degree of independence and ability to consent.

Project SHINE is currently centered in New York City (NYC), where there is a large racially, culturally, and economically diverse population of people with ID. A multidisciplinary network of experts within the ID and sexual health fields as well as an Advisory Board of self-advocates with ID and caregivers (i.e., parents, guardians, kinship carers) of adolescents and young adults with ID carry out the work of Project SHINE. The SHINE Network consists of 14 members from organizations that provide supportive services for people with disabilities, sexual health care and education, disability rights, and advocacy. The SHINE Advisory Board supports the Network in establishing project goals and holds the Network accountable for achieving them.

## Methods

Project SHINE research staff conducted exploratory, formative research to inform the development of innovative new sexuality education learning tools. This included focus group discussions with youth with ID and parents of youth with ID, findings from which are reported elsewhere (Colarossi et al., 2022). Findings from the survey and focus groups with professionals are the focus of this manuscript. With disability support professionals, we used a cross-sectional mixed-methods approach starting with a survey and followed 4 months later by focus groups to explore gaps in the quantitative data and obtain in-depth information related to needed teaching tools and methods. The full Project SHINE Network provided multiple perspectives for measure creation and data interpretation. Transdisciplinary and mixed-method approaches have been recommended as a best practice in health care research, especially with underserved groups (Harper et al., 2008), and further input was provided by Project SHINE Advisory Board members (people with ID, caregivers, and people working in the disability field).

### Participants and Data Collection

The sampling frame for the study was direct service providers, managers, and administrators employed by four Project SHINE Network organizations serving people with ID (*N* = 6091). Sterling IRB approved all study procedures and waived the requirement for signed consent; participants were provided with a study information document to review prior to data collection and could participate or decline without anyone from their organization knowing their choice. Inclusion criteria were: age 18 years or older; employed by an agency serving youth with ID and/or their caregivers; working in a role involving direct services to, or management of services for, youth ages 16–24 years old with mild to moderate ID (which could be concurrent with other diagnoses) or their caregivers; written and verbal fluency in English; and for focus group participants only, able to participate in a private space via a virtual meeting platform by both audio and video and willing to be part of a recorded meeting.

For the survey, we recruited all organizational staff via an email invitation with an electronic survey link. The survey incentive was a $20 electronic gift card to the first 200 respondents. The survey link was open for 1 week in January 2021, and in that time, 757 staff responded, 655 met eligibility criteria, and 632 completed the survey (10.4% participation rate).

We purposively recruited focus group participants from two SHINE Network organizations, seeking participants in three categories: (1) licensed/credentialed direct service professionals (social workers, nurses, teachers), (2) non-licensed direct service providers (case managers, supportive care specialists, residential care line staff), and (3) program/agency administrators. We recruited from two organizations because these represented the largest number of direct service providers and served the most economically disadvantaged youth. Network members connected prospective participants in these categories with the research team via an email recruitment letter specifying dates and times of four planned 90-min groups (held in April and May of 2021). Interested staff completed a brief electronic registration form to confirm their consent and provide professional role and personal demographic information. Staff who registered then received an official invitation to join the virtual meeting on the Zoom platform. In total, there were 36 participants in 4 focus groups and group sizes ranged from 7 to 11 participants; each received a $40 electronic gift card for an online retailer as an incentive.

### Measures

Instructions to survey and focus group participants were to answer all questions about “youth” with the definition of “adolescents and young adults approximately 16–24 years old with any diagnosis of intellectual and/or developmental disability (also called neurodevelopmental disorder) who are able to communicate (verbally or otherwise) with or without supports.” Therefore, all measures described below using the term “youth” should be understood in this way. The SHINE Network and Advisory Board provided input into the final measures.

*Demographic* data we collected were gender (woman, man, transgender, nonbinary/nonconforming, and other with a write-in option), race/ethnicity (African American or Black, Asian, Hispanic or Latino/Indigenous American or Alaska Native, Native Hawaiian or Pacific Islander, White, and other with a write-in option), and job roles of administrators, licensed professionals (social workers, medical providers, teachers), and non-licensed direct care providers (case managers, youth development specialists, supportive care services).

*Attitudes* about providing SRH information to youth with ID were measured on the survey using four items with which participants were asked to indicate their level of agreement on a four-point Likert scale (1 = *strongly disagree* to 4 = *strongly agree*). Items included: “It is best to wait for young people to ask questions about sexuality rather than bring up the topic myself”; “Access to sexual and reproductive health information and materials (e.g., condoms, books) will encourage sexual behavior.” The items were mean scored into a scale with good internal reliability (Cronbach’s alpha = .76).

The survey also asked, “How important is it that youth receive ongoing, medically accurate information about each of the following topics?” Participants answered using a four-point Likert scale (1 = *not at all* to 4 = *very important*). Eleven topics (e.g., sexually transmitted infection testing and treatment, birth control, sexual development and hygiene) were mean scored into a scale with excellent internal reliability (Cronbach’s alpha = .91)*.*

The focus group guide included a discussion question, “what kinds of information about sexual and reproductive health do you think are most important for young people ages 16-24 with mild to moderate ID?” Probes focused on three broad areas: (1) sexual development and anatomy, (2) reproduction, birth control, and access to SRH services, and (3) intimate relationships, consent, and bodily autonomy.

*Professional preparedness* was measured on the survey using the stem: “In general, how prepared are you to talk about the following topics with youth?” Eleven topics (e.g., sexually transmitted infection testing and treatment, birth control, sexual development and hygiene) were rated on four-point Likert scales (1 = *not at all* to 4 = *very prepared*). A mean scale was created that had excellent internal reliability (Alpha = .94). The focus group guide included a question, “What kinds of things do professionals need to learn to help them support the sexual health of people with intellectual disabilities?” Probes included communication skills (e.g., opening conversations; answering tricky questions); health care referrals, specific SRH content areas; and how to use teaching aids such as condom demonstrations, videos, and engagement activities.

*Needs in the field* were measured with five survey items using the stem, “What do you professionally need to support the sexual and reproductive health of youth?” The response options were 1 = *not at all needed*, 2 = *minimally needed*, 3 = *somewhat needed*, and 4 = *very much needed*. The items were as follows: professional training; standard sex ed curriculum specifically for youth with ID; my agency’s support for integrating sexual and reproductive health into job roles and expectations; specialized educational tools for youth with ID such as books, pictures, videos, activities/exercises; and other support (with a write-in option).

The focus group guide had a discussion question, “What kinds of educational tools would be helpful for youth, which they could use by themselves or in one-on-one conversations with parents/caregivers or professionals or even during workshop-based interventions?” Probes included videos, games, digital and web-based content, workbooks and other print materials, and hands-on models or toolkits.

*Communication practices* were measured with survey questions about frequency of communicating SRH information (“In the last year, have you provided sexual and reproductive health information to youth as part of your job?”), with equivalent questions about providing information to youth and to parents or guardians for their youth. Participants who responded to this initial yes/no question in the affirmative were asked to indicate how they had done so using the following response options: *with all youth that I serve*; *on an as-needed basis related to a specific need or question*; or *by making referrals to other staff but not discussing these topics directly*. In the focus groups, we asked, “What kinds of things do professionals need to learn to help them support the sexual health of youth with intellectual disabilities?” with specific probing about communication skills.

### Analyses

Descriptive, bi- and multi-variate analyses were conducted with the survey data to describe the sample as a whole and determine differences between groups within the sample such as by job role. Verbatim transcripts of focus groups were created from the Zoom closed captioning transcript feature, then downloaded and cleaned for spelling by research staff. Responses were labeled with deidentified participant ID numbers to facilitate comparison of responses. Research staff were not part of the organizations where the participants worked and separated personally identifying information from the transcripts and coding. We used an applied thematic analysis approach to coding the transcripts (Guest et al., 2012), aided by Dedoose software. This approach involved an initial round of structural coding followed by a second round to identify emergent themes. For the structural coding, two different coders (one assigned to each transcript) segmented text using a total of nine a priori codes aligned with the structure of the discussion guides, and which were outlined in a codebook detailing a code name associated with a particular discussion topic, a brief definition, and scenarios for use. We then further analyzed the segments associated with five structural codes (professional attitudes, professional needs, SRH topics for youth education, teaching methods and tools for youth, and parent educational needs) to identify emergent themes. Identifying repetition—by multiple participants within a single group as well as across groups—was our primary strategy to define themes. After an initial reading of all the transcript segments (exported from Dedoose into summary documents), two coders met to discuss their perspectives on emergent themes and identify a set of thematic codes. Finally, the coders used Dedoose to apply the agreed-upon thematic codes to the transcript segments.

## Results

### Participant Characteristics

Information on the participants’ gender, race and ethnicity, and professional roles is summarized in Table 1. The survey participants were diverse in terms of their racial and ethnic identities and in their professional roles; however, majorities identified as women and as non-licensed direct service providers. Relative to the survey participants, higher proportions of focus group participants identified as white and were licensed professionals.

**Table 1:** Participant characteristics

**Characteristic**	**Focus groups** ***N*** **= 36**	**Surveys** ***N*** **= 632**
**Gender**		
Woman Man Transgender Nonbinary	30 (83%) 5 (14%) 0 1 (3%)	518 (82%) 97 (15%) 7 (1%) 10 (2%)
**Race/ethnicity**		
Black Asian Indigenous American, Alaskan, or Hawaiian Latino/a or Hispanic, any race White Multiracial Unknown/missing	6 (17%) 2 (6%) 0 4 (11%) 21 (58%) 1 (3%) 2 (6%)	160 (25%) 35(6%) 4 (1%) 153 (24%) 261 (41%) 9 (1%) 10 (2%)
**Primary professional role**		
Administrator/manager Non-licensed direct service provider Licensed professionals	7 (19%) 6 (17%) 23 (64%)	88 (14%) 330 (52%) 214 (34%)

### Attitudes

Survey results show attitudes about providing SRH information were moderately positive on average with some variation by job role (see Table 2). ANOVA and post hoc test results showed significant differences in attitudes by job role such that non-licensed providers reported somewhat less positive attitudes (*M* = 2.04, *SD* = .68) than did administrators (*M* = 1.85, *SD* = .63) and licensed providers (*M* = 1.78, *SD* = .62; *F* = 10.67, *p* < .001). (Items were worded so that greater agreement with the statements indicates less supportive attitudes toward the SRH needs of youth with ID.) The perceived importance of discussing SRH topics was rated more positively overall (*M* = 3.87, *SD* = .31) with no significant differences by job role.

**Table 2:** Survey results by professional role

**Survey item**	**Scale Mean (SD)**				**Test Statistics**		
	**Overall** **N = 632**	**Administrators** ***n* = 88**	**Licensed professionals** ***n* = 214**	**Non-licensed professionals** ***n* = 330**	***F***	***df***	***p***
Attitudes about SRH education for youth^a^	1.93 (.66)	1.85 (.63)	1.78 (.62)	2.04 (.68)	10.67	2	<.001
Importance of SRH topics for youth education^b^	3.87 (.31)	3.86 (.33)	3.89 (.30)	3.86 (.31)	.22	2	.804
Prepared to provide SRH information to youth^c^	3.18 (.62)	3.13 (.55)	3.21 (.59)	3.18 (.65)	.51	2	.599
Professional needs^d^							
Parent workshops	3.72 (.52)	3.76 (.46)	3.77 (.47)	3.68 (.57)	2.26	2	.105
Specialized sex ed curriculum for youth with ID	3.58 (.67)	3.44 (.70)	3.63 (.63)	3.58 (.68)	2.33	2	.098
Professional training	3.52 (.67)	3.40 (.72)	3.51 (.65)	3.55 (.66)	1.89	2	.153
Sex ed tools (e.g., books, pictures, videos, activities/exercises)	3.49 (.70)	3.39 (.74)	3.53 (.63)	3.49 (.73)	1.32	2	.269
Agency support for integrating SRH into job roles and expectations	3.36 (.76)	3.29 (.72)	3.35 (.76)	3.39 (.74)	.52	2	.595

	**Item N (%)**				**Test Statistics**		
	**Overall** **N = 632**	**Administrators** ***n* = 88**	**Licensed professionals** ***n* = 214**	**Non-licensed professionals** ***n* = 330**	**χ**^**2**^	***df***	***p***
Communication about SRH with youth in the last year					22.47	4	<.001
Not at all or refer elsewhere	457 (72.3%)	76 (86.4%)	132 (61.7%)	249 (75.5%)			
Only as needed for a specific issue or question	154 (24.4%)	11 (12.5%)	72 (33.6%)	71 (21.5%)			
Routinely with all youth	21 (3.3%)	1 (1.1%)	10 (4.7%)	10 (3.0%)			
Communication about SRH with parents in the last year					42.19	4	<.001
Not at all or refer elsewhere	502 (79.4%)	78 (88.6%)	139 (65.0%)	285 (86.4%)			
Only as needed for a specific issue or question	108 (17.1%)	9 (10.2%)	63 (29.4%)	36 (10.9%)			
Routinely with all youth	22 (3.5%)	1 (1.1%)	12 (5.6%)	9 (2.7%)			

^a^1 = strongly positive, 4 = strongly negative

^b^1 = not at all important, 4 = very important

^c^1 = not at all prepared, 4 = very prepared

^d^1 = not at all, 2 = minimal need, 3 = somewhat needed, 4 = very much needed

In the qualitative data, three themes emerged. These are described and supported with exemplary quotes in Table 3. The first theme was that both professionals and the parents/caregivers they work with commonly view youth with ID as non-sexual, which in turn impacts the SRH information professionals provide to youth. For example, one licensed professional described how parents of youth with ID react to conversations about sexuality: “It’s like, ‘oh no, our kids are never going to engage in sexual health, our kids have disabilities.’” The second emergent theme was that providers need to learn how to interact with youth about SRH in unbiased, fact-based, non-judgmental ways. The third theme was discomfort talking about SRH and normalizing sexuality for both youth and professionals. One non-licensed professional discussed this discomfort and how they work through it by displaying visual cues in their work area that can be conversation starters, saying, “Maybe they’re initially not comfortable or they don’t want to have the conversation but as they’re looking around, [they see] I have a yellow sticker that I got at a training that says, ‘We care about your sexual health. Got a question? Ask me.’”

**Table 3:** Qualitative results

**Structural code**	**Theme**	**Exemplary quotes**
Attitudes about providing SRH information to youth with ID	1. Youth with ID are non-sexual, which impacts the SRH information they receive	“Everyone that is supposed to be helpful and supportive treats the people with ID as children who aren’t meant to have these feelings instead of treating them like growing individuals.” – Administrator “I think education to parents, caregivers and providers about what it's like to have ID and experience the normal human emotions of sexual desires. Explaining how to openly discuss these topics and share ways desires can be met for them. Others who care for the individuals with ID tend to have a hard time understanding that sex and desire for sexual relationships are normal for everyone, including those with ID. It seems providers and caregivers are wanting to stop individuals with ID from exploring their sexuality and desires.” – Non-licensed provider
	2. Providers need to learn how to have unbiased, fact-based, non- judgmental interactions with youth	“I think when talking about sexuality, it's important that we convey to the person: it's okay we are sexual beings. In so many times we don't say, ‘you're a sexual being, you can be sexual,’ and while we're working with that person, be non-judgmental. I think it's important for whoever's doing the assessment and/or working with the group for their facial expression to be neutral. And that's something we have to practice, so that the person will open up to us. And, so we have our own prejudices, so we have to be aware of that.” – Licensed provider
	3. Discomfort prevents communication	“Now we can have the greatest materials and greatest games and cubes and conversations but sometimes we struggle even opening the conversation because they [youth] think it's not important, or they don't know or are embarrassed, “Why are you talking to me about this?”, even though I preface that this is my job actually to do this, they're still not comfortable sometimes.” – Licensed provider
Preparedness to provide SRH information to youth	1. Lack of training	“[We need] For all professionals to be trained in how to talk about these subjects. People with ID want to have these types of conversations with people they are familiar with and trust; not with some "sex professional." The professional sex educators should train [our agency’s] staff so the staff can work with people effectively.” – Non-licensed provider
		“I think people need training on how to have conversations about sexual health with this age group [youth 16–24 years old with ID]. I really don't think there's enough mandatory training. There's like an assumption, I think, that people who are even social workers, who have their master's degree, know how to have these conversations. Even though that's not even one of the classes [that is offered in social work school]… it's not even something our agency had where our staff are trained, because there is a little bit of an assumption that everyone knows how to have those conversations, and I don't agree that that is the case.” – Administrator/manager
	2. Lack of awareness of tools or information that do exist	“I’m going to speak with the perspective, both as a parent and as a professional. I think that there's not enough awareness in the field of what materials do exist because I think there are some really good materials out there that I've been introduced to as a parent for my younger neurotypical kids, like how to introduce them to their developing bodies and how babies are made and things like that, in ways that I think could actually be used really easily also with some older kids that might be a little bit more neurotypical. But I don't think that there's a lot of dissemination of that information and awareness of what does exist out there. It's not marketed well, I think, to professionals.” – Licensed provider “I'm having a hard time finding the proper tools to do a group on sexual health well…it's really hard to find the tools to talk to these kids about sexual reproduction, sexual health, good touch bad touch, proper names for your body parts.” – Licensed provider
Professional needs	1. Accessible teaching tools	“And when [youth] come in [to a residential treatment setting], they have a question of well, ‘How does this thing happen?’ or in some various things and it’s either I have the information or a lot of times it's Google. Like I'll go on Google and we'll look it up and luckily I think I have a good understanding of what sites are reputable versus, you know, like we're not going to WebMD, we're going more to Mayo Clinic or National Institute of Health. But then I have to translate it into more accessible language for them and explain what these terms mean which can be challenging sometimes, so knowing virtual material or websites that are accessible [is needed] because that's a lot of the work we do and a lot of where the kids are looking anyway.” – Non-licensed provider “With the people that I work with, visuals should be very interactive and hands-on, so they can be able to maybe kind of touch the material, feel the material, and something visual so they can feel something very tactile for my folks so they can see what it is, feel it, and just be in the whole experience. I think that would be a very good learning tool for many of our guys as well too, instead of having something on the board or just reading out something but to something they can feel and touch as well too.” – Non-licensed provider
	2. Agency policies and guidelines	“Us as social workers that are in the homes every day, with behavior specialists that are in the homes, week by week, social workers that we definitely need these tools, and I also think we need to do it uniformly. Foster parents need to be saying the same things, the medical department needs to be saying the same things.” – Licensed provider “I mean in terms of having a clear understanding of where the agency's standpoint is on whatever the issue might be that comes up, so that you don't go into a phone call with a parent without the information that you need, if that makes sense. Because I wouldn't want to make any type of false promises, or provide parents any incorrect information from where the agency comes from. We're a family-oriented, family-run agency and oftentimes families very much do have a sway on some of the more ethical dilemmas that we encounter.” – Non-licensed provider “In my department, in my team specifically, I think it just comes up when it comes up, and then those conversations are had with whoever is around, and we don't always, not from what I've seen in the agency as a whole, we don't really have a super structured way of doing it.” – Administrator “As a staff member, you sort of have to jump through a couple of extra hoops sometimes to make sure that [sexuality education] is going to fit your billable service that you're meant to be providing at that point. There are people who are just supposed to be getting services about employment, and yeah, you can you can make a case for that, if something sexually inappropriate might be happening in a workplace, but the service is not designed to run that way from jump. Even if it might be in that person's best interest…So it can be hard to find a place to do those things, even if you know that that's what the person you're working with really needs.” – Non-licensed provider
SRH communication with youth	1. Ambivalence about who should provide information – dedicated staff or everyone should be trained	“I think we often rely also on who has the person's trust, so we have had times when it's not the nurses, not the psychologist, it's the staff member who kind of, it's the habit or whatever or sometimes things happen organically, like conversation comes up and you're not going to say like, wait, hold your thought let me go get the nurse.” – Non- licensed provider “We always kind of – I think we just don't have enough designated staff to really be the go-to for that kind of thing… that's one of the needs that I think we probably have as an agency, is designated people who really respond to that stuff, who can kind of counsel and have those discussions and talk to the families and be their port of call.” – Non-licensed provider “I’d say in my experience, there's been a lot of relying on our medical team to step in to have these conversations. I think there's still needs to be a lot of work on getting staff that are working with this population across the board all being comfortable having these conversations, but that speaks to all sorts of people's own personal preferences and how comfortable they are talking about this.” – Administrator “Working in the residential [setting], I think one of the challenges we often find is finding a certified sex therapist. If we had more certified therapists available to provide the type of training that some of the men and women may need, that may be helpful, because a lot of times you find out, it's a lot of the issues or concerns come from sexual frustration. And so, you need a trained professional to help train the men and women how to relieve their frustrations.” – Non-licensed provider
	2. Reactionary communications are the status quo	“I think that most of the time there's maybe one person of each gender in each office, who is the go-to with [sexuality] issues, but so much of the time they're just being contacted after a problem has presented itself. You know, there was a period where if someone who had acted sexually inappropriate [in the program], I would definitely get a call about it and then we would have a conversation, but I probably wouldn't necessarily do that until there was a reason. And at that point we sort of already failed.” – Non-licensed provider “Sometimes the people we support get trained [in sexual harassment], but usually as a reactionary thing…it’s usually something happens and then it’s like, ‘Oh, we should probably tell them why that wasn’t appropriate.’” – Non-licensed provider
SRH communication with parents	1. Primary role of parents for providing SRH information	“Usually, I think that conversation is held with parents and their PCP whenever that conversation comes about. I can definitely say I have teenagers in my caseload and I haven't really had those type of conversations with them. And it brings me back, so okay, when do we have those conversations and do we have them [at all]? And unless they bring them up or unless the parents bring them up, how do we address them? Because we normally don't. I mean, in my perspective professionally and even personally, I'll leave that up to the parents to do them.” – Licensed provider “And so we think that parents are afraid of it. Yeah, they're afraid of it, and we [staff] don't talk about it… …[So,] it's not going to go anywhere.” – Licensed provider
	2. Need for professionals to help parents be able to understand the SRH of youth	“Not all of us here are in foster care or involved with foster care youth and for prevention, it is different. We don't have the requirements in the same way. And we're rarely seeing kids apart from their parent. So that we need to be thinking about conversations that can happen with the parent there.” – Licensed provider “Our work is a lot with these caregivers, trying to help them make sure that they're giving their child the information that they need when we're not there supporting, or supporting their child to get that information, because they can't do as much on their own, like maybe normally developing children, like going to Planned Parenthood or going somewhere on their own. So, I agree we need to learn how to have these conversations with the caregivers present, and we interact with these kids more than the medical team. I do think we want that information being disseminated as often as possible. The whole time I've been here I've just been thinking about caregivers, like just more training for caregivers, then almost for the children, because those kids need support.” – Non-licensed provider

### Preparedness

Survey results showed that staff feel between “somewhat” and “very” prepared to provide SRH information to youth (*M* = 3.18, *SD* = .62; see Table 2). ANOVA analyses showed no significant differences by job role. In the qualitative data, however, there was far more discussion about a lack of preparedness among professionals and no focus group participants mentioned feeling sufficiently prepared.

Two themes emerged from the qualitative data (Table 3): (1) Providers lack training in how to discuss SRH with youth in accurate and unbiased ways, and (2) Providers lack awareness of medically accurate SRH information and tools that they could use in their work. These themes are conveyed in a quotation from a non-licensed professional who said, “It is just all so complex and for me as a practitioner, it’s really difficult because I’m just like, ‘Oh gosh, where do I start and how do I start?’”

### Needs in the Field

Survey respondents rated parent (*M* = 3.72, *SD* = .52) and youth (*M* = 3.58, *SD* =.67) workshop curricula as most greatly needed to support the SRH of youth, followed closely by professional training (*M* = 3.52, *SD* = .67), educational tools (*M* = 3.49, *SD* = .70), and agency support to integrate SRH into job roles and expectations (*M* = 3.36, *SD* = .76; Table 2). Two themes related to needs emerged from the qualitative data (Table 3). First, participants expressed the need for accessible teaching tools, with videos and hands-on toolkits being mentioned repeatedly. One non-licensed provider explained, “With the people that I work with, visuals should be very interactive and hands-on, so they can be able to maybe kind of touch the material, feel the material, and something visual so they can feel something very tactile for my folks.” Contrary to the quantitative data, standardized curriculum was not mentioned with any notable frequency. The second theme was the need for organizational policies and guidelines for communicating about SRH with youth and guardians. One non-licensed professional referred to this as “clarity from the higher-ups,” while others described that without a clear set of organizational policies, staff may adopt idiosyncratic communication practices.

### Communication Practices

Best practice in SRH is to have routine, preventative conversations (Goldfarb & Lieberman, 2021). Survey data (Table 2) show that very few providers (about 3%) report having routine, universal conversations about SRH with youth or their guardians. Some reported having conversations on an as-needed basis only to address specific issues or questions from youth (24%) or guardians (17%). The vast majority reported not having any conversations with youth (72%) or guardians (79%) but referred them to someone else (like a nurse or educator). Results of chi-square analyses to compare communication practices by job role (Table 2) showed that licensed providers were significantly more likely to discuss SRH on an as-needed basis with youth and guardians than were non-licensed providers and administrators.

To further explore the findings on communication practices, we conducted ANOVA analyses to compare whether provider attitudes and preparedness differed across these three communication categories (results are shown in Table 4). Professionals’ attitudes were not associated with talking to youth about SRH, but preparedness was. Preparedness was significantly higher among professionals who talked about SRH with youth routinely (*M* = 3.64, *SD* = .44) or as needed (*M* = 3.38, *SD* = .50), compared to those who did not discuss SRH at all with youth in the past year (*M* = 3.09, *SD* = .64; *F* = 19.54, *p* < .001). Both attitudes and preparedness were significantly associated with professionals’ communication with guardians about SRH. Attitudes were more negative among those who routinely communicated with guardians (*M* = 2.29, *SD* = .96), compared to those who had such conversations only as needed (*M* = 1.67, *SD* = .57) or not at all (*M* = 1.97, *SD* = .65; *F* = 12.39, *p* < .001). Professionals who reported routine (*M* = 3.47, *SD* = .61) or as-needed (*M* = 3.34, *SD* = .53) conversations with guardians reported a significantly higher level of preparedness than those who reported no recent communication with guardians (*M* = 3.13, *SD* = .63; *F* = 7.60, *p* <.001).

**Table 4:** ANOVA results of attitudes and preparedness by communication with youth and parents

	**Talked with all youth *M* (SD)**	**On an as-needed basis *M* (SD)**	**Did not discuss *M* (SD)**	***F***
**Attitudes**	1.95 (.76)	1.92 (.72)	1.93 (.34)	.04
**Prepared**	3.64 (.44)	3.38 (.50)	3.09 (.64)	19.54*

	**Talked with all parents** ***M*** **(*SD*)**	**On an as-needed basis** ***M*** **(*SD*)**	**Did not discuss** ***M*** **(*SD*)**	***F***
**Attitudes**	2.29 (.96)	1.67 (.57)	1.97 (.65)	12.39*
**Prepared**	3.47 (.61)	3.34 (.53)	3.13 (.63)	7.60*

^*^p < .001. Prepared Bonferroni post hoc test show professionals who talked with all youth and who did so on an as-needed basis significantly different from did not discuss. Prepared Bonferroni post hoc test show talked with all parents and on as-needed basis significantly different from did not discuss. Attitudes Bonferroni post hoc test show talked with all parents significantly different from on an as-needed basis and did not discuss

Related to communicating SRH information to youth, two themes emerged from the qualitative data. The first was that opinions were mixed about who should be responsible for talking with youth: some believed that everyone should be trained and able to provide SRH information, while others believed that only specific professionals (e.g., sex educators and medical providers) should have it within their job role to do so. The second theme was that reactionary, incident-driven conversations about sexuality (particularly issues of sexual boundaries, consent, and sexual harassment) are the norm. As one licensed professional described, “A lot of the conversations are incident-driven…maybe there will be some sexual interaction that happens that you become aware of and then it’s like, ‘Oh, this conversation needs to happen,’ rather than being more proactive.” This theme aligns with survey findings that most SRH communication is happening in response to a specific need.

Finally, there were qualitative themes related to the roles of guardians and professionals related to communicating about SRH with youth. Professionals said guardians should be the primary sexuality educators for their children, but some shared concerns that this happens rarely in practice. The second theme was that professionals have a role to play in supporting guardians of youth with ID to help them communicate directly with youth in their care.

## Discussion

This study adds to the literature on the role of disability support professionals in facilitating access to sexual health information, education, and care for adolescents and young adults with mild to moderate ID. We triangulated data from an online survey of a diverse group of New York-based professionals (*N* = 632), slightly more than half of whom were non-licensed direct care providers such as case managers and youth development specialists, and four 90-min focus groups (*N* = 36) in which about two-thirds of participants were licensed professionals such as social workers and psychologists. Our findings show that both individual (attitudes) and environmental (agency norms and policies, availability of accessible teaching tools) factors may influence the way professionals provide SRH information to youth.

Survey results indicated that professionals in this study held generally supportive attitudes toward providing SRH information to youth with ID. We also found significantly more supportive attitudes among licensed professionals and administrators in comparison to non-licensed professionals. Our findings are consistent with other research showing heterogeneity among professionals based on role characteristics (Bazzo et al., 2007). Professionals expressed concern that their colleagues, as well as guardians, hold the erroneous belief that youth with ID are not sexual and that this belief acts as a barrier to the provision of SRH resources. Professionals in a study by Schmidt and colleagues (2021) similarly identified parents’ beliefs that their children are not ready for or interested in relationships as a barrier to sex education. Our qualitative findings further suggest that training to increase professionals’ knowledge and comfort level with SRH content could foster more supportive attitudes toward sexuality for youth with ID. Quantitatively, professionals who reported conversations with guardians about SRH in the past year reported feeling more prepared for such conversations, but surprisingly, also held less positive attitudes about supporting SRH needs for youth with ID compared to professionals who never spoke with guardians or did so only as needed. This could reflect beliefs that guardians, rather than professionals, are more suited to providing sexuality-related information.

Survey participants rated themselves moderately prepared to provide SRH information to youth, with perceived preparedness positively correlated with the frequency of communicating SRH information to youth. Very few survey participants (3%), however, reported having routine conversations about SRH with youth or their guardians. This finding aligns with what was shared in the focus groups: that communication about sexuality tends to be incident-driven rather than proactive and routine (similar to findings by Schaafsma et al., 2014 and Schmidt et al., 2021). Focus group participants also spoke of needing training and informational/educational resources to increase their preparedness, but opinions were mixed about the value of universal training for all professionals versus designating specific staff roles as sexuality resource persons.

Professionals in this study expressed high levels of interest in additional resources to support the SRH of youth with ID, similar to others’ research (Abbott & Howarth, 2007; McConkey & Ryan, 2001). Survey findings indicated that educational curricula were most greatly needed, followed by professional training. In the focus groups, accessible teaching tools such as videos and hands-on teaching tools, as well as organizational policies and guidelines about providing SRH information and care emerged as the greatest needs. This difference may be due to a greater proportion of licensed professionals and program managers in focus groups compared to more non-licensed service providers completing the survey.

### Limitations and Strengths

We did not use a probability sampling approach to recruit survey participants, and thus it is possible that professionals with greater interest in or stronger feelings about the topic were more likely to participate. To address this possible limitation, we notified all staff within the participating organizations and offered an incentive to motivate broad participation. Recruiting professionals via their employers also raises the possibility of response bias. To minimize this possibility, the research team (positioned outside any of the organizations from which professionals were recruited) communicated with prospective participants directly and kept the identities of focus group participants confidential. Survey responses were anonymous, and follow-up to those who provided contact information (via a separate survey) to receive the incentive was handled by the research team. We note that the focus group participant sample did not have the same racial/ethnic and role diversity as the survey sample—a larger proportion of focus group participants identified as white and were licensed professionals. Focus group feedback may thus be more reflective of the perspectives of professionals who carry both racial privilege and positional power within their organizations. Finally, while findings from New York-based organizations may not generalize to other geographic contexts, our robust survey sample size (representing 10% of the workforce at the participating organizations), recruitment of participants from multiple organizations, and use of mixed methods are notable strengths.

### Implications for Practice

Professionals need opportunities to develop basic knowledge of SRH content as well as relevant, accessible resources for SRH information and care. Training opportunities should build communication skills and understanding of best practices for communicating factual SRH information, addressing questions, and making referrals, and doing so proactively. It is likely that routine, consistent communication about SRH is less apt to be shaming or negative than communication that is strictly reactive. As we heard in the focus groups, organizations may wish to pursue models of universal basic training for all staff or more intensive training for a select group of more specialized staff, but we recommend that organizations using the latter model minimally train all staff on how to connect youth to their internally designated champions or “SRH experts.”

Professional development is reinforced when organizations have policies and practices that are guided by a statement of rights for people with ID that includes informed choices about sexual expression and access to SRH care. Professionals completing the survey indicated a need for agency support of their efforts to communicate and educate about SRH. As Schmidt et al. (2021) heard from professionals working with youth with intellectual and developmental disabilities, professionals hesitate to provide in-depth SRH information when they do not perceive institutional support that would protect them from allegations of improper behavior. Further, medically accurate, developmentally appropriate educational tools (e.g., pamphlets, books, teaching aids) and referral resources must be available in service settings. Technical assistance to organizational leaders has been a useful supplement in other contexts such as after-school programs and foster care agencies (Colarossi et al., 2014, 2019). Such assistance has included, for example, development of organizational sexuality guiding principles, action planning to sustain learnings from staff training, and provision of tools and supplies for sex education.

Combined with data collected from parents and youth with ID (described in the literature and in their participation in this study as reported elsewhere), the findings point to the need for tools and/or a toolkit that can be used with multiple stakeholder groups—youth, guardians and people working in the disability field. It is also important that there be continued education that conveys that youth with disabilities are sexual beings and deserving of information about their bodies and sexuality. Everyone within a youth’s social system has a role for ensuring they are opening the door to information and care, not serving as a barrier or assuming others will take on this role. Tools need to support proactive conversations and be engaging so that they are accessible in both methodology (e.g., clear and concrete language and multiple teaching methods) and in use (e.g., being fun and approachable to address discomfort).

The SHINE Network and Advisory Board is using this data to create a multi-modal toolkit to meet these needs, with plans to test prototypes in 2022–23. The toolkit will feature six equitable teaching components that will work independently or together for use by youth with ID and their support networks in a variety of settings. A committee of the SHINE Network, Advisory Board, and youth with ID aged 16–24 convene weekly to use the formative research findings to design prototypes of tools that will be further tested for acceptability, feasibility, and comprehension.

## Data Availability

Data are not yet publicly available, but may be requested from the authors.
